# Rab GTPases: The Key Players in the Molecular Pathway of Parkinson’s Disease

**DOI:** 10.3389/fncel.2017.00081

**Published:** 2017-03-28

**Authors:** Meng-meng Shi, Chang-he Shi, Yu-ming Xu

**Affiliations:** Department of Neurology, The first affiliated Hospital, Zhengzhou UniversityZhengzhou, China

**Keywords:** Parkinson’s disease, Rab GTPases, α-synuclein, *LRRK2*, *PINK1* Parkin, *TMEM230*, *Rab39b*

## Abstract

Parkinson’s disease (PD) is a progressive movement disorder with multiple non-motor symptoms. Although family genetic mutations only account for a small proportion of the cases, these mutations have provided several lines of evidence for the pathogenesis of PD, such as mitochondrial dysfunction, protein misfolding and aggregation, and the impaired autophagy-lysosome system. Recently, vesicle trafficking defect has emerged as a potential pathogenesis underlying this disease. Rab GTPases, serving as the core regulators of cellular membrane dynamics, may play an important role in the molecular pathway of PD through the complex interplay with numerous factors and PD-related genes. This might shed new light on the potential therapeutic strategies. In this review, we emphasize the important role of Rab GTPases in vesicle trafficking and summarize the interactions between Rab GTPases and different PD-related genes.

## Introduction

Parkinson’s disease (PD) is the second most prevalent chronic neurodegenerative disorder of aging, clinically characterized by motor symptoms including resting tremor, muscle rigidity, bradykinesia, postural instability and various non-motor symptoms (Shi et al., 2016). The most evident pathological features are the progressive degeneration of dopaminergic neurons and axonal projections in the substantia nigra and the wide spreading of eosinophilic Lewy bodies whose cardinal component is α-synuclein detected in some surviving neurons (Goedert et al., 2013).

**FIGURE 1:**
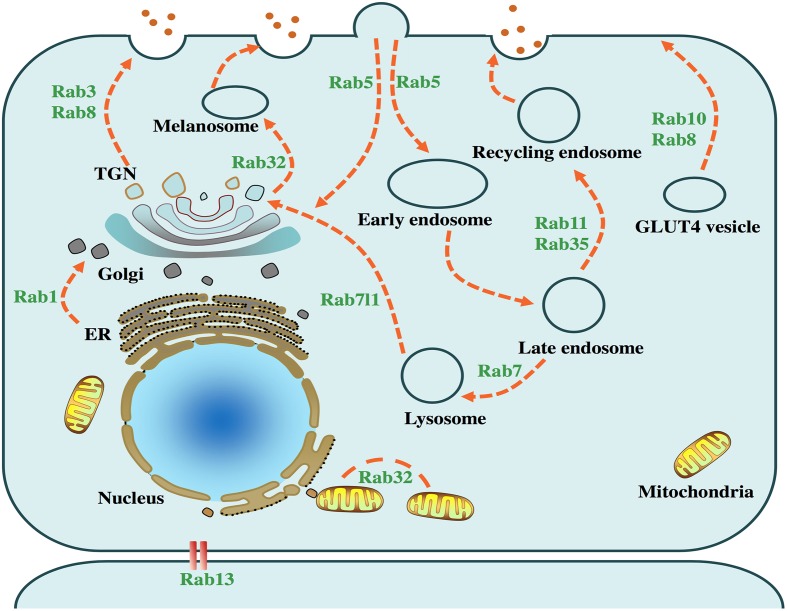
**Physiological functions of PD-related Rabs**. Intracellular vesicle trafficking pathways and several related Rab GTPases are presented in the above cell model. Rab1 facilitates endoplasmic reticulum (ER) to Golgi trafficking. Rab3, accompanied by Rab8, mediates the trafficking from *trans*-Golgi network (TGN) to the plasma membrane. Rab5, serving as a key factor in the early endosome formation, is required for the transport of clathrin-dependent endosomes. Rab7 mediates the fusion of late endosomes and lysosomes, which plays an important role in the autophagy-lysosome pathway. Rab8 and Rab10 facilitate trafficking of glucose transporter type 4 (GLUT4) to the plasma membrane. Rab13 regulates the junctions among epithelial cells. Rab11 and Rab35 regulate the recycling endosome trafficking. Rab7l1 plays a role in the trafficking from lysosome to TGN. Rab32 mediates the fission of mitochondria and the trafficking from TGN to melanosome.

The etiology underlying the development of PD remains elusive. To date, approximate 18 genes have been identified as the genetic causes for familiar PD, which have provided critical clues for the pathogenesis of the disease (Atashrazm and Dzamko, 2016). Recently, accumulating genetic discoveries have revealed the association between vesicle trafficking and PD (Zimprich et al., 2011; Edvardson et al., 2012; Wilson et al., 2014). Disruption of the cellular vesicle trafficking results in the impaired degradation of certain proteins and further leads to the abnormal protein aggregation, which exerts a toxic effect on neurons (Mazzulli et al., 2016). Rab GTPases (Rabs) perform the basic functions in intracellular trafficking events (**Figure 1**). Moreover, a series of recent studies have revealed that certain Rabs are involved in the modulation of α-synuclein. Impairment of these proteins have been reported to be one of the rare causes for inherited early onset PD (Wilson et al., 2014; Lesage et al., 2015; Mata et al., 2015; Shi et al., 2016). These new findings provide a novel insight into the molecular pathogenesis of PD. Moreover, the pathogenic mechanisms of different PD-related genes (*SNCA*, *LRRK2*, *PINK1, Parkin* and *TMEM230*) may share a converging molecular pathway, and Rabs may serve as potential modulators in this pathway. In this review, we summarize the physiological functions of Rabs and their interactions with multiple PD-related genes (**Table 1**).

**Table 1:** Parkinson’s disease-related Rab GTPases.

Rab GTPases	Localization	Physiological function	Related genes	Relevant Pathogenesis
Rab1	ER, *cis*-Golgi, intermediate compartment	ER-Golgi	*SNCA*	Rescue the toxicity induced by aberrant α-synuclein.
Rab3	Secretory vesicles, PM	Exocytosis, neurotransmitter release	*SNCA, LRRK2*	Colocalize with α-synuclein and regulate its distribution. Substrate of LRRK2-mediated phosphorylation.
Rab5	PM, CCVs, early endosome	Endocytosis, early endosome fusion	*SNCA, LRRK2, TMEM230*	Colocalize with α-synuclein, LRRK2 and TMEM230. Together with LRRK2 regulate synaptic vesicle endocytosis.
Rab7	Late endosome, lysosomes,	Late endosome to lysosome	*SNCA, LRRK2, Parkin, TMEM230*	Colocalize with α-synuclein and TMEM230. Reverse the disturbance induced by *LRRK2* defect *in vivo* and *in vitro*. Involve the *Parkin*-mediated mitophagy.
Rab8	TGN, GLUT4-positive vesicles	TGN-PM transport, GLUT4 trafficking	*SNCA, LRRK2, PINK1*	Interact with α-synuclein and alleviate the toxicity induced by aberrant α-synuclein. Substrate of LRRK2/PINK1-mediated phosphorylation.
Rab10	Golgi, GLUT4-positive vesicles	Exocytosis, TGN/RE to PM, GLUT4 trafficking	*LRRK2*	Substrate of LRRK2 -mediated phosphorylation.
Rab11	Golgi, recycling endosome, early endosome	TGN/RE to PM	*SNCA, TMEM230*	Interact with α-synuclein and modulate its secretion. Colocalize with TMEM230.
Rab13	Tight junctions, TGN	Junctions among epithelial cells	*SNCA*	Alleviate the toxicity induced by aberrant α-synuclein.
Rab7L1	Lysosomes	Lysosome to TGN	*LRRK2*	Together with LRRK2 involve the trafficking from lysosome to TGN.
Rab32	Mitochondria, melanosomes	TGN to melanosome, mitochondrial fission	*LRRK2*	Control LRRK2-related late endosomal events.
Rab35	PM, clathrin-coated pits, recycling endosomes	RE to PM	*SNCA*	Promote the aggregation and secretion of A53T α-synuclein.
Rab39b	Golgi	Unknown	*SNCA*	Modulate the localization of α-synuclein.

*ER, endoplasmic reticulum; PM, plasma membrane; CCV, clathrin-coated vesicles; TGN, trans-Golgi network; GLUT4, Glucose transporter type 4; RE, recycling endosome*.

## Rabs and their Roles in the Vesicle Trafficking Pathway

Rabs are the largest subfamily of Ras-like GTPases with more than 60 members in the human genome and 11 members in the yeast, serving as molecular “switches” in vesicle trafficking (Pereira-Leal and Seabra, 2001). Generally consisting of 200 amino acids, Rabs are widely distributed across the eukaryotic cells. They perform the essential functions in the formation, maturation, transport, tethering and fusion of vesicles, regulating the interactions among the major organelles and maintaining the cellular homeostasis (Binotti et al., 2016). Some structural features of the primary structure contribute to the specific interactions among membranes (Pereira-Leal and Seabra, 2000; Lipatova et al., 2015). The GTP-binding regions are highly conserved in evolution and make Rabs present in inactive GDP-bound and active GTP-bound forms. The CAAX boxes usually contain two cysteine residues and facilitate the attachment of Rabs to the membrane after prenylation. Located upstream at CAAX boxes, the hypervariable region may enable targeting Rabs to the specific effectors. Additionally, the Rab family (RabF) motifs (RabFl∼RabF5) serve to differentiate Rabs from other members of the Ras superfamily; the Rab subfamily (RabSF) sequences play an important part in the identification of various subfamilies (Pereira-Leal and Seabra, 2000). Some studies indicate that apart from the diversity of C-terminal, RabF and RabSF are also important for the specific interactions with target effectors and membranes (Ali and Seabra, 2005). The GTPase fold contains a six-stranded sheet and five α helices which are interconnected by 10 loops. Different forms of nucleotide-bound Rabs present distinct conformation involving the switch I and switch II regions (Stroupe and Brunger, 2000). Through the cycle between active and inactive states, Rabs achieve the function as molecular “switches.” The new inactive GDP-bound form interacts with Rab escort protein (REP) and is delivered to a geranylgeranyl transferase (GGTase) (Alexandrov et al., 1994). After prenylation, the Rab GDP dissociation inhibitor (GDI) recognizes the GDP-bound Rab and regulates the specific insertion to the membrane with the assistance of a GDI dissociation factor (GDF) (Sivars et al., 2003). Once the Rab interacts with the target membrane, it transforms from the GDP-bound form to the GTP-bound form, a reaction that is catalyzed by guanine nucleotide exchange factor (GEF) (Delprato et al., 2004). The active GTP-bound form is recognized by the effector proteins including sorting adaptors, tethering complexes, motor proteins and various enzymes (Hutagalung and Novick, 2011). Followed by hydrolysis which is stimulated by the GTPase activating protein (GAP), the trafficking event comes to an end (Pan et al., 2006).

Vesicle trafficking in the clathrin-dependent or clathrin-independent manner is the core process for the cellular membrane dynamics. Numerous Rabs are involved in the budding, uncoating, motility and fusion of vesicles via interaction with the relevant effectors. Once a Rab stimulates the association between the sorting adaptor and the distinct receptor, the cargo is shipped into a budding vesicle. Rab9, whose effector is TIP47, facilitates the interaction between TIP47 and cytoplasmic terminal of mannose-6-phosphate receptors (M6PRs) during the formation of vesicles and maintains the recycle of M6PRs from the late endosome to the trans-Golgi network (TGN) (Aivazian et al., 2006). Coated vesicles account for most of the membrane trafficking, and the coats are removed to allow the fusion of vesicles. The assembly polypeptide 2 (AP2) is crucial for the recruitment of clathrin, of which the μ2 subunit interacts with the cargo after phosphorylation by μ2 kinase (Jackson et al., 2003). Rab5 is involved in the regulation of vesicle uncoating via either the removal of μ2 kinase or the turnover of phosphatidylinositol-4, 5-biphosphate [PI (4, 5) P2] (Semerdjieva et al., 2008). To validate the correct delivery of vesicles to the relevant membranes, it is necessary for Rabs to mediate vesicle trafficking along a series of motor proteins and microtubules. For example, myosin Va is linked to Rab27 by melanophilin working as an adaptor protein, and then delivers the relevant vesicles to the pericellular matrix to allow melanocytes to achieve their physiological functions (Wu et al., 2002). To ensure the specificity of membrane fusion, Rabs may cooperate with tethering factors and mediate interactions between vesicles and the relevant membrane. As a result of the overlapping binding site of endosome antigen 1 (EEA1) for Rab5 and syntaxin-6, Rabs may interact directly with SNAREs and regulate the docking and fusion with target membranes (Simonsen et al., 1999).

## Rabs Interact with α-Synuclein and Modulate its Distribution

α-Synuclein is a small protein encoded by *SNCA*, whose role in the pathogenesis of PD has been highly debated over the years. Initially identified as a presynaptic protein, α-synuclein may play an important role in endo- or exocytosis of synaptic vesicles (Hansen et al., 2011). Recent studies revealed that α-synuclein diffused among the cells via a prion-like transmission, and the presence of extracellular α-synuclein challenged the previous notion indicating that α-synuclein was limited to the cytoplasm (El-Agnaf et al., 2003; Lee et al., 2006; Desplats et al., 2009; Hansen et al., 2011). As for lacking a signal sequence, part of extracellular α-synuclein has been confirmed to translocate across the membrane via the exocytosis pathway, which relies on calcium rather than the conventional manner, and the process is promoted under the stressing conditions (Lee et al., 2005; Jang et al., 2010; Emmanouilidou and Vekrellis, 2016). Additionally, extracellular α-synuclein can also return to the neurons via endocytosis or the clathrin-dependent manner (Liu et al., 2007; Lee et al., 2008). All of the above studies have shed new light on the role of α-synuclein in the pathogenesis of PD and indicate the possible interactions between α-synuclein and Rabs which play a critical role in modulating the vesicle trafficking.

A large number of studies have made efforts to clarify the interactions between Rabs and α-synuclein, and this field can be elucidated from two different perspectives. On the one hand, by a train of immunofluorescence, co-immunoprecipitation and other strategies, certain Rabs (Rab3a, Rab5, Rab8, Rab7, and Rab11a) have been confirmed to interact with α-synuclein in different models and protect cells from the toxicity induced by the mutation or over-expression of α-synuclein (Hasegawa et al., 2011; Chutna et al., 2014). α-Synuclein accumulation is liable to collapse endoplasmic reticulum-Golgi trafficking during the process of tethering or docking in a manner of dose and time dependence, which can be alleviated by overexpression of Ypt1/ Rab1 which helps the COPII vesicles uncoating in yeast or dopaminergic neurons (Cooper et al., 2006; Gitler et al., 2008). The interaction between Rab1 and α-synuclein may be mediated by prenylated Rab acceptor protein (PRA1) which serves as a GDF for Rab1 (Lee et al., 2011). Using a *Drosophila* model, Breda et al. (2015) revealed that overexpression of Rab11 colocalizing with α-synuclein in intracellular inclusions could significantly reverse the synaptic potentiation at the neuromuscular junction due to the increase of synaptic vesicle size. Through a shRNA-based screen, some Rabs (Rab8b, Rab11a, and Rab13) have been identified as modulators of α-Synuclein in living cells and reduced the toxicity induced by aggregated α-synuclein via secretion enhancement (Goncalves et al., 2016). Rab8, playing a role in the post-Golgi trafficking, binded with the C terminal of α-synuclein, which was confirmed by the nuclear magnetic resonance spectroscopy. In cell models, Rab8 suppressed the toxicity caused by mutation or overexpression of α-synuclein (Yin et al., 2014). On the other hand, Rabs may be involved in regulating the recycling and distribution of α-synuclein, which opens a new window for new therapeutic strategies. Apart from degradation by the lysosomal pathway, the internalized extracellular α-synuclein might be partly secreted from cells via exocytosis, which might be facilitated by Rab11 (Liu et al., 2009; Chutna et al., 2014). Chutna et al. (2014) conducted a further study that showed the co-localization of Rab11 and α-synuclein *in vivo*. In addition, they also found that Rab11 rescued the α-synuclein aggregation and cytotoxicity. However, the mechanism underlying how Rab11 regulates the secretion of α-synuclein is still disputed (Chutna et al., 2014). Besides, the downregulation of the novel disease-causing gene Rab39b results in the dysregulation of α-synuclein homeostasis, which will be extensively elucidated below (Wilson et al., 2014). As a protein regulating synaptic vesicles, Rab3a not only has been confirmed to have a close association with α-synuclein but also may lead to the re-distribution of α-synuclein via a conformation change (Chen et al., 2013). Recently, Rab35 was identified as a potential biomarker in the serum for the differential diagnosis and progression of PD. Besides, the functional study also suggested that overexpression of Rab35 led to increased aggregation and secretion of aberrant α-synuclein (Chiu et al., 2016). Above all, these lines of evidence suggest that the α-synuclein pathology propagation may partly attribute to the dysregulation of Rabs.

## The Interactions between Rabs and Different PD-Related Genes

### Leucine-Rich Repeat Kinase 2

Leucine-rich repeat kinase 2 (LRRK2, also known as PARK8) is a multi-domain 280 kDa protein characterized with functional GTPase and kinase domains (Kang and Marto, 2016). Most PD-specific mutations of *LRRK2* occur in the afore-mentioned regions and lead to autosomal recessive and several sporadic PD, which hints at the importance of these enzymes in the pathogenesis (Funayama et al., 2005; Gilks et al., 2005; Kachergus et al., 2005; Tan et al., 2007; Martin et al., 2014). LRRK2 is involved in diverse cellular molecular events, such as synaptic vesicle dynamic, mitochondrial function and autophagy (Plowey et al., 2008; Shin et al., 2008; Wang et al., 2012). To support the role of *LRRK2* in vesicle trafficking, there are a few lines of evidence suggesting the complex interactions between *LRRK2* and certain Rabs.

Since the identification of the first *LRRK2*-related Rab GTPase, Rab5b, involved in synaptic vesicle endocytosis, effort has been made to investigate the novel interactive GTPases (Shin et al., 2008). Lrrk, a homolog of human LRRK2 in *Drosophila*, interacted strikingly with Rab7 and regulated the localization of lysosome (Dodson et al., 2012). Besides, this study also linked the pathogenesis underlying LRRK2 G2019S to the disturbance of Rab7-mediated lysosome positioning (Dodson et al., 2012). Another study also identified that the impaired trafficking from early to late endosomes induced by the mutation of *LRRK2* could be alleviated by coexpression of Rab7, which deepened the understanding of the interaction between LRRK2 and Rab7 (Gomez-Suaga et al., 2014). Using the transcriptomic approach, LRRK2 has been reported to bind with Rab7l1 whose polymorphism confers the reduced risk of PD at the Golgi apparatus, and the degeneration induced by G2019S could be rescued by the overexpression of the Rab7l1 in the *Drosophila* dopamine neurons (Gan-Or et al., 2012). Furthermore, in the case of *LRRK2* mutation or *Rab7l1* knockdown, the retromer pathway components levels including VPS35 appeared reduced, which suggested that LRRK2 or Rab7l1 might be involved in the retromer pathway (MacLeod et al., 2013). Besides, Steger et al. used the approach of high-resolution quantitative mass spectrometry and combined it with other approaches to identify the substrates of LRRK2. However, only two sites met the strict criteria: pSer935 in LRRK2 and pThr73 of the small GTPase Rab10. In the following study, they also found the phosphorylation of some Rabs (Rab3, Rab8), which contained a Thr at the site equivalent to T73-Rab10 *in vitro* and *in vivo*. Interestingly, all of the above sites were in the switch II region which mediated the cycle of GDP/GTP for the interaction with the cellular membrane. Overexpression of *LRRK2*, to some extent, might lower the affinity of some Rabs to the GDIs and caused the abnormal insertion to the membrane and disruption of the vesicle trafficking (Steger et al., 2016). It appears that Rabs work as a downstream regulator of LRRK2 to achieve their role in vesicle trafficking. Conversely, in another study Waschbusch et al. (2014) found that Rab32 might directly interact with the amino terminal of endogenous LRRK2 and influenced LRRK2-related late endosomal events. Taken together, the physiological function of LRRK2 in vesicle trafficking is closely associated with Rabs and the dysfunction of either of the components may result in a defect in vesicle dynamics, which could ultimately lead to PD.

### PTEN-Induced Kinase 1/Parkin

Loss-of-function mutations in the PTEN-induced kinase 1 (*PINK1*) and RBR E3 ubiquitin protein ligase (*Parkin*) usually lead to autosomal recessive PD (Kitada et al., 1998; Valente et al., 2004). It has been reported that PINK1 worked as the upstream regulator of Parkin in a common pathway and was linked to the mitochondrial quality control. Once mitochondrial dysfunction is detected by PINK1, Parkin is recruited to ubiquitinate the damaged mitochondrial and clear them up via autophagy (Narendra et al., 2008, 2010; Ziviani et al., 2010). PD patients with *PINK1* or *Parkin* defects usually exhibit similar phenotypes. However, Dave et al. (2014) observed that knockout of *Parkin* in rats did not cause so serious neurodegeneration as *PINK1*, which implicated that additional proteins might share a common pathway (Khan et al., 2002). A few investigations suggest that Rabs are candidate proteins to serve in downstream steps of the mitophagy pathway. Lai et al. (2015) conducted a SILAC-based phosphoproteomic screening to identify the novel substrates of PINK1. They found that Ser111 of three Rab GTPases (Rab8a, Rab8b, and Rab13) were phosphorylated in a PINK-dependent and Parkin-independent manner. Phosphorylation of this site in Rabs might impair the interaction with the respective GEF, which thereby disrupted the activation of Rabs (Lai et al., 2015). Rab7 as well as its GAP TBC1D15 and TBC1D17 have been reported to be involved in the Parkin-mediated mitophagy. Both TBC1D15 and TBCD17 played an important role in the process of autophagosome formation and prevented the membrane from Rab7-mediated inflation (Yamano et al., 2014).

### Transmembrane-Protein 230

Transmembrane-protein 230 (*TMEM230*) gene presumably encodes a transmembrane protein with elusive physiological localization and function. The latest study by Deng et al. (2016) investigating on a large North American family has revealed that TMEM230 was a disease-causing gene of PD with typical pathological features of Lewy bodies. To characterize the human TMEM230 distribution and function, a series of confocal microscopy experiments were conducted. The results showed that vesicle structures characterized with TMEM230 were predominantly located in the perinucleus region and colocalized with the markers of synaptic vesicles. Given the multiple roles of Rabs in vesicle trafficking, they also found interactions between TMEM230 and Rab5a, Rab7 or Rab11a, respectively. These findings, in turn, suggested that TMEM230 might play a role in the process of vesicle formation and trafficking; the impairment of this protein might lead to pathological features in neurons in patients with PD (Deng et al., 2016).

### Rab39b

*Rab39b*, first isolated from the human fetal brain, encodes a neuron-specific protein with putative functions in the synapse formation and maintenance (Cheng et al., 2002). Mapping to the human chromosome Xq28, loss-of-function mutations or increased dosage of *Rab39b* were confirmed to be linked to the molecular basis of X-linked mental retardation (Giannandrea et al., 2010). To explore the mechanism underlying Rab39b-related cognitive deficits, there was evidence suggesting that Rab39b regulated the alpha-amino-3-hydroxy-5-methyl-4-isoxazole propionic acid receptor (AMPAR) arrangement in the case of interaction with the PDZ domain of protein interacting with C-kinase 1 (PICK1), which was important for synaptic plasticity (Mignogna et al., 2015). Genetic analysis of Australian and Wisconsin kindreds first identified that the defect in *Rab39b* caused pathologically defined PD. Following functional studies revealed that altered *Rab39b* was rapidly metabolized through the proteasome pathway and might dysregulate the localization of α-synuclein (Wilson et al., 2014). Based on this novel breakthrough, a large number of studies were conducted to further investigate the genetic contribution of *Rab39b* to the pathogenesis of PD (Lesage et al., 2015; Mata et al., 2015; Guldner et al., 2016). Recently, our team also identified a novel mutation of *Rab39b* (c.536 dup A) in a family, of which the patients displayed juvenile parkinsonism and mental retardation syndromes. Moreover, the brain magnetic resonance imaging (MRI) and computed tomography (CT) scans of both patients showed calcification in the basal ganglia, which was never reported in the previous cases (Shi et al., 2016). According to the latest study, the complex formed by C9ORF72, WDR41 and SMCR8 worked as a GEF for Rab39b as well as Rab8a, which suggested that Rab39b might be involved in the autophagy regulation (Sellier et al., 2016). Albeit with constant studies focusing on the characterization of *Rab39b* molecular functions, the mechanisms of genetic defects still need to be explored.

## Conclusion

Over the years, many researchers have paid much attention to exploring the pathogenesis of PD to halt or slow the progression of PD and identify effective therapeutic strategies. Different hypothesizes have been proposed, such as mitochondrial dysfunction, protein misfolding and aggregation, and impaired autophagy-lysosome system. Recently, the focus has shifted to vesicle trafficking. In this review, we emphasized the importance of the functions of Rabs and their potential interplay with different PD-related genes. Rabs perform an essential role in vesicle trafficking and cellular homeostasis maintenance. Not only are Rabs closely associated with α-synuclein-mediated pathological process but they also interact with many genes whose mutations or polymorphisms lead to the development of PD. It is plausible that Rabs could be regarded as novel biomarkers or therapeutic targets before the clinical manifestation of PD. However, the role of Rabs in the molecular pathway of PD remains elusive. More functional studies should be conducted to reveal the interplay between Rabs and different effectors.

## Ethics Statement

This study did not involve any ethical issues. The studies about MC1R and Rab39b received approval from the institutional ethics committee of Zhengzhou University.

## Author Contributions

The conception or design of the work: M-mS, C-hS, and Y-mX. Drafting the work or revising it: M-mS, C-hS and Y-mX. Final approval of the version to be published: M-mS, C-hS, and Y-mX. Agreement to be accountable for all aspects of the work: M-mS, C-hS, and Y-mX.

## Conflict of Interest Statement

The authors declare that the research was conducted in the absence of any commercial or financial relationships that could be construed as a potential conflict of interest.

## References

[B1] Aivazian D. Serrano R. L. Pfeffer S. (2006). TIP47 is a key effector for Rab9 localization. *J. Cell Biol.* 173 917–926. 10.1083/jcb.200510010 16769818 PMC2063917

[B2] Alexandrov K. Horiuchi H. Steele-Mortimer O. Seabra M. C. Zerial M. (1994). Rab escort protein-1 is a multifunctional protein that accompanies newly prenylated rab proteins to their target membranes. *EMBO J.* 13 5262–5273. 7957092 10.1002/j.1460-2075.1994.tb06860.x PMC395482

[B3] Ali B. R. Seabra M. C. (2005). Targeting of Rab GTPases to cellular membranes. *Biochem. Soc. Trans.* 33(Pt 4), 652–656. 10.1042/BST0330652 16042566

[B4] Atashrazm F. Dzamko N. (2016). LRRK2 inhibitors and their potential in the treatment of Parkinson’s disease: current perspectives. *Clin. Pharmacol.* 8 177–189. 10.2147/CPAA.S102191 27799832 PMC5076802

[B5] Binotti B. Jahn R. Chua J. J. (2016). Functions of Rab proteins at presynaptic sites. *Cells* 5 E7. 10.3390/cells5010007 PMC4810092 26861397

[B6] Breda C. Nugent M. L. Estranero J. G. Kyriacou C. P. Outeiro T. F. Steinert J. R. (2015). Rab11 modulates alpha-synuclein-mediated defects in synaptic transmission and behaviour. *Hum. Mol. Genet.* 24 1077–1091. 10.1093/hmg/ddu521 25305083 PMC4986550

[B7] Chen R. H. Wislet-Gendebien S. Samuel F. Visanji N. P. Zhang G. Marsilio D. (2013). alpha-Synuclein membrane association is regulated by the Rab3a recycling machinery and presynaptic activity. *J. Biol. Chem.* 288 7438–7449. 10.1074/jbc.M112.439497 23344955 PMC3597785

[B8] Cheng H. Ma Y. Ni X. Jiang M. Guo L. Ying K. (2002). Isolation and characterization of a human novel RAB (RAB39B) gene. *Cytogenet. Genome. Res.* 97 72–75. 10.1159/000064047 12438742

[B9] Chiu C. C. Yeh T. H. Lai S. C. Weng Y. H. Huang Y. C. Cheng Y. C. (2016). Increased Rab35 expression is a potential biomarker and implicated in the pathogenesis of Parkinson’s disease. *Oncotarget* 7 54215–54227. 10.18632/oncotarget.11090 27509057 PMC5342336

[B10] Chutna O. Goncalves S. Villar-Pique A. Guerreiro P. Marijanovic Z. Mendes T. (2014). The small GTPase Rab11 co-localizes with alpha-synuclein in intracellular inclusions and modulates its aggregation, secretion and toxicity. *Hum. Mol. Genet.* 23 6732–6745. 10.1093/hmg/ddu391 25092884

[B11] Cooper A. A. Gitler A. D. Cashikar A. Haynes C. M. Hill K. J. Bhullar B. (2006). Alpha-synuclein blocks ER-Golgi traffic and Rab1 rescues neuron loss in Parkinson’s models. *Science* 313 324–328. 10.1126/science.1129462 16794039 PMC1983366

[B12] Dave K. D. De Silva S. Sheth N. P. Ramboz S. Beck M. J. Quang C. (2014). Phenotypic characterization of recessive gene knockout rat models of Parkinson’s disease. *Neurobiol. Dis.* 70 190–203. 10.1016/j.nbd.2014.06.009 24969022

[B13] Delprato A. Merithew E. Lambright D. G. (2004). Structure, exchange determinants, and family-wide rab specificity of the tandem helical bundle and Vps9 domains of Rabex-5. *Cell* 118 607–617. 10.1016/j.cell.2004.08.009 15339665

[B14] Deng H. X. Shi Y. Yang Y. Ahmeti K. B. Miller N. Huang C. (2016). Identification of TMEM230 mutations in familial Parkinson’s disease. *Nat. Genet.* 48 733–739. 10.1038/ng.3589 27270108 PMC6047531

[B15] Desplats P. Lee H. J. Bae E. J. Patrick C. Rockenstein E. Crews L. (2009). Inclusion formation and neuronal cell death through neuron-to-neuron transmission of alpha-synuclein. *Proc. Natl. Acad. Sci. U.S.A.* 106 13010–13015. 10.1073/pnas.0903691106 19651612 PMC2722313

[B16] Dodson M. W. Zhang T. Jiang C. Chen S. Guo M. (2012). Roles of the *Drosophila* LRRK2 homolog in Rab7-dependent lysosomal positioning. *Hum. Mol. Genet.* 21 1350–1363. 10.1093/hmg/ddr573 22171073 PMC3284123

[B17] Edvardson S. Cinnamon Y. Ta-Shma A. Shaag A. Yim Y. I. Zenvirt S. (2012). A deleterious mutation in DNAJC6 encoding the neuronal-specific clathrin-uncoating co-chaperone auxilin, is associated with juvenile parkinsonism. *PLoS ONE* 7:e36458 10.1371/journal.pone.0036458 PMC3341348 22563501

[B18] El-Agnaf O. M. Salem S. A. Paleologou K. E. Cooper L. J. Fullwood N. J. Gibson M. J. (2003). Alpha-synuclein implicated in Parkinson’s disease is present in extracellular biological fluids, including human plasma. *FASEB J.* 17 1945–1947. 10.1096/fj.03-0098fje 14519670

[B19] Emmanouilidou E. Vekrellis K. (2016). Exocytosis and spreading of normal and Aberrant alpha-Synuclein. *Brain Pathol.* 26 398–403. 10.1111/bpa.12373 26940375 PMC8029167

[B20] Funayama M. Hasegawa K. Ohta E. Kawashima N. Komiyama M. Kowa H. (2005). An LRRK2 mutation as a cause for the parkinsonism in the original PARK8 family. *Ann. Neurol.* 57 918–921. 10.1002/ana.20484 15880653

[B21] Gan-Or Z. Bar-Shira A. Dahary D. Mirelman A. Kedmi M. Gurevich T. (2012). Association of sequence alterations in the putative promoter of RAB7L1 with a reduced parkinson disease risk. *Arch. Neurol.* 69 105–110. 10.1001/archneurol.2011.924 22232350

[B22] Giannandrea M. Bianchi V. Mignogna M. L. Sirri A. Carrabino S. D’Elia E. (2010). Mutations in the small GTPase gene RAB39B are responsible for X-linked mental retardation associated with autism, epilepsy, and macrocephaly. *Am. J. Hum. Genet.* 86 185–195. 10.1016/j.ajhg.2010.01.011 20159109 PMC2820185

[B23] Gilks W. P. Abou-Sleiman P. M. Gandhi S. Jain S. Singleton A. Lees A. J. (2005). A common LRRK2 mutation in idiopathic Parkinson’s disease. *Lancet* 365 415–416. 10.1016/S0140-6736(05)17830-1 15680457

[B24] Gitler A. D. Bevis B. J. Shorter J. Strathearn K. E. Hamamichi S. Su L. J. (2008). The Parkinson’s disease protein alpha-synuclein disrupts cellular Rab homeostasis. *Proc. Natl. Acad. Sci. U.S.A.* 105 145–150. 10.1073/pnas.0710685105 18162536 PMC2224176

[B25] Goedert M. Spillantini M. G. Del Tredici K. Braak H. (2013). 100 years of Lewy pathology. *Nat. Rev. Neurol.* 9 13–24. 10.1038/nrneurol.2012.242 23183883

[B26] Gomez-Suaga P. Rivero-Rios P. Fdez E. Blanca Ramirez M. Ferrer I. Aiastui A. (2014). LRRK2 delays degradative receptor trafficking by impeding late endosomal budding through decreasing Rab7 activity. *Hum. Mol. Genet.* 23 6779–6796. 10.1093/hmg/ddu395 25080504

[B27] Goncalves S. A. Macedo D. Raquel H. Simoes P. D. Giorgini F. Ramalho J. S. (2016). shRNA-based screen identifies endocytic recycling pathway components that act as genetic modifiers of Alpha-synuclein aggregation, secretion and toxicity. *PLoS Genet.* 12:e1005995 10.1371/journal.pgen.1005995 PMC4849646 27123591

[B28] Guldner M. Schulte C. Hauser A. K. Gasser T. Brockmann K. (2016). Broad clinical phenotype in Parkinsonism associated with a base pair deletion in RAB39B and additional POLG variant. *Parkinsonism. Relat. Disord.* 31 148–150. 10.1016/j.parkreldis.2016.07.005 27448726

[B29] Hansen C. Angot E. Bergstrom A. L. Steiner J. A. Pieri L. Paul G. (2011). alpha-Synuclein propagates from mouse brain to grafted dopaminergic neurons and seeds aggregation in cultured human cells. *J. Clin. Invest.* 121 715–725. 10.1172/JCI43366 21245577 PMC3026723

[B30] Hasegawa T. Konno M. Baba T. Sugeno N. Kikuchi A. Kobayashi M. (2011). The AAA-ATPase VPS4 regulates extracellular secretion and lysosomal targeting of alpha-synuclein. *PLoS ONE* 6:e29460 10.1371/journal.pone.0029460 PMC3245276 22216284

[B31] Hutagalung A. H. Novick P. J. (2011). Role of Rab GTPases in membrane traffic and cell physiology. *Physiol. Rev.* 91 119–149. 10.1152/physrev.00059.2009 21248164 PMC3710122

[B32] Jackson A. P. Flett A. Smythe C. Hufton L. Wettey F. R. Smythe E. (2003). Clathrin promotes incorporation of cargo into coated pits by activation of the AP2 adaptor micro2 kinase. *J. Cell Biol.* 163 231–236. 10.1083/jcb.200304079 14581451 PMC2173513

[B33] Jang A. Lee H. J. Suk J. E. Jung J. W. Kim K. P. Lee S. J. (2010). Non-classical exocytosis of alpha-synuclein is sensitive to folding states and promoted under stress conditions. *J. Neurochem.* 113 1263–1274. 10.1111/j.1471-4159.2010.06695.x 20345754

[B34] Kachergus J. Mata I. F. Hulihan M. Taylor J. P. Lincoln S. Aasly J. (2005). Identification of a novel LRRK2 mutation linked to autosomal dominant parkinsonism: evidence of a common founder across European populations. *Am. J. Hum. Genet.* 76 672–680. 10.1086/429256 15726496 PMC1199304

[B35] Kang U. B. Marto J. A. (2016). Leucine-rich repeat kinase 2 (LRRK2) and Parkinson’s disease. *Proteomics* 10.1002/pmic.201600092 [Epub ahead of print]. 27723254

[B36] Khan N. L. Valente E. M. Bentivoglio A. R. Wood N. W. Albanese A. Brooks D. J. (2002). Clinical and subclinical dopaminergic dysfunction in PARK6-linked parkinsonism: an 18F-dopa PET study. *Ann. Neurol.* 52 849–853. 10.1002/ana.10417 12447943

[B37] Kitada T. Asakawa S. Hattori N. Matsumine H. Yamamura Y. Minoshima S. (1998). Mutations in the parkin gene cause autosomal recessive juvenile parkinsonism. *Nature* 392 605–608. 10.1038/33416 9560156

[B38] Lai Y. C. Kondapalli C. Lehneck R. Procter J. B. Dill B. D. Woodroof H. I. (2015). Phosphoproteomic screening identifies Rab GTPases as novel downstream targets of PINK1. *EMBO J.* 34 2840–2861. 10.15252/embj.201591593 26471730 PMC4654935

[B39] Lee H. J. Kang S. J. Lee K. Im H. (2011). Human alpha-synuclein modulates vesicle trafficking through its interaction with prenylated Rab acceptor protein 1. *Biochem. Biophys. Res. Commun.* 412 526–531. 10.1016/j.bbrc.2011.07.028 21798244

[B40] Lee H. J. Patel S. Lee S. J. (2005). Intravesicular localization and exocytosis of alpha-synuclein and its aggregates. *J. Neurosci.* 25 6016–6024. 10.1523/JNEUROSCI.0692-05.2005 15976091 PMC6724798

[B41] Lee H. J. Suk J. E. Bae E. J. Lee J. H. Paik S. R. Lee S. J. (2008). Assembly-dependent endocytosis and clearance of extracellular alpha-synuclein. *Int. J. Biochem. Cell Biol.* 40 1835–1849. 10.1016/j.biocel.2008.01.017 18291704

[B42] Lee P. H. Lee G. Park H. J. Bang O. Y. Joo I. S. Huh K. (2006). The plasma alpha-synuclein levels in patients with Parkinson’s disease and multiple system atrophy. *J. Neural. Transm. (Vienna)* 113 1435–1439. 10.1007/s00702-005-0427-9 16465458

[B43] Lesage S. Bras J. Cormier-Dequaire F. Condroyer C. Nicolas A. Darwent L. (2015). Loss-of-function mutations in RAB39B are associated with typical early-onset Parkinson disease. *Neurol. Genet.* 1 e9. 10.1212/NXG.0000000000000009 PMC4821081 27066548

[B44] Lipatova Z. Hain A. U. Nazarko V. Y. Segev N. (2015). Ypt/Rab GTPases: principles learned from yeast. *Crit. Rev. Biochem. Mol. Biol.* 50 203–211. 10.3109/10409238.2015.1014023 25702751 PMC4722870

[B45] Liu J. Zhang J. P. Shi M. Quinn T. Bradner J. Beyer R. (2009). Rab11a and HSP90 regulate recycling of extracellular alpha-synuclein. *J. Neurosci.* 29 1480–1485. 10.1523/JNEUROSCI.6202-08.2009 19193894 PMC3241981

[B46] Liu J. Zhou Y. Wang Y. Fong H. Murray T. M. Zhang J. (2007). Identification of proteins involved in microglial endocytosis of alpha-synuclein. *J. Proteome Res.* 6 3614–3627. 10.1021/pr0701512 17676786

[B47] MacLeod D. A. Rhinn H. Kuwahara T. Zolin A. Di Paolo G. McCabe B. D. (2013). RAB7L1 interacts with LRRK2 to modify intraneuronal protein sorting and Parkinson’s disease risk. *Neuron* 77 425–439. 10.1016/j.neuron.2012.11.033 23395371 PMC3646583

[B48] Martin I. Kim J. W. Dawson V. L. Dawson T. M. (2014). LRRK2 pathobiology in Parkinson’s disease. *J. Neurochem.* 131 554–565. 10.1111/jnc.12949 25251388 PMC4237709

[B49] Mata I. F. Jang Y. Kim C. H. Hanna D. S. Dorschner M. O. Samii A. (2015). The RAB39B p.*G192R* mutation causes X-linked dominant Parkinson’s disease. *Mol. Neurodegener.* 10 50 10.1186/s13024-015-0045-4 PMC4581468 26399558

[B50] Mazzulli J. R. Zunke F. Isacson O. Studer L. Krainc D. (2016). alpha-Synuclein-induced lysosomal dysfunction occurs through disruptions in protein trafficking in human midbrain synucleinopathy models. *Proc. Natl. Acad. Sci. U.S.A.* 113 1931–1936. 10.1073/pnas.1520335113 26839413 PMC4763774

[B51] Mignogna M. L. Giannandrea M. Gurgone A. Fanelli F. Raimondi F. Mapelli L. (2015). The intellectual disability protein RAB39B selectively regulates GluA2 trafficking to determine synaptic AMPAR composition. *Nat Commun* 6 6504 10.1038/ncomms7504 PMC4383008 25784538

[B52] Narendra D. Tanaka A. Suen D. F. Youle R. J. (2008). Parkin is recruited selectively to impaired mitochondria and promotes their autophagy. *J. Cell Biol.* 183 795–803. 10.1083/jcb.200809125 19029340 PMC2592826

[B53] Narendra D. P. Jin S. M. Tanaka A. Suen D. F. Gautier C. A. Shen J. (2010). PINK1 is selectively stabilized on impaired mitochondria to activate Parkin. *PLoS Biol.* 8:e1000298 10.1371/journal.pbio.1000298 PMC2811155 20126261

[B54] Pan X. Eathiraj S. Munson M. Lambright D. G. (2006). TBC-domain GAPs for Rab GTPases accelerate GTP hydrolysis by a dual-finger mechanism. *Nature* 442 303–306. 10.1038/nature04847 16855591

[B55] Pereira-Leal J. B. Seabra M. C. (2000). The mammalian Rab family of small GTPases: definition of family and subfamily sequence motifs suggests a mechanism for functional specificity in the Ras superfamily. *J. Mol. Biol.* 301 1077–1087. 10.1006/jmbi.2000.4010 10966806

[B56] Pereira-Leal J. B. Seabra M. C. (2001). Evolution of the Rab family of small GTP-binding proteins. *J. Mol. Biol.* 313 889–901. 10.1006/jmbi.2001.5072 11697911

[B57] Plowey E. D. Cherra S. J. III Liu Y. J. Chu C. T. (2008). Role of autophagy in G2019S-LRRK2-associated neurite shortening in differentiated SH-SY5Y cells. *J. Neurochem.* 105 1048–1056. 10.1111/j.1471-4159.2008.05217.x 18182054 PMC2361385

[B58] Sellier C. Campanari M. L. Julie Corbier C. Gaucherot A. Kolb-Cheynel I. Oulad-Abdelghani M. (2016). Loss of C9ORF72 impairs autophagy and synergizes with polyQ Ataxin-2 to induce motor neuron dysfunction and cell death. *EMBO J.* 35 1276–1297. 10.15252/embj.201593350 27103069 PMC4910533

[B59] Semerdjieva S. Shortt B. Maxwell E. Singh S. Fonarev P. Hansen J. (2008). Coordinated regulation of AP2 uncoating from clathrin-coated vesicles by rab5 and hRME-6. *J. Cell Biol.* 183 499–511. 10.1083/jcb.200806016 18981233 PMC2575790

[B60] Shi C. H. Zhang S. Y. Yang Z. H. Yang J. Shang D. D. Mao C. Y. (2016). A novel RAB39B gene mutation in X-linked juvenile parkinsonism with basal ganglia calcification. *Mov. Disord.* 31 1905–1909. 10.1002/mds.26828 27943471

[B61] Shin N. Jeong H. Kwon J. Heo H. Y. Kwon J. J. Yun H. J. (2008). LRRK2 regulates synaptic vesicle endocytosis. *Exp. Cell Res.* 314 2055–2065. 10.1016/j.yexcr.2008.02.015 18445495

[B62] Simonsen A. Gaullier J. M. D’Arrigo A. Stenmark H. (1999). The Rab5 effector EEA1 interacts directly with syntaxin-6. *J. Biol. Chem.* 274 28857–28860. 10.1074/jbc.274.41.28857 10506127

[B63] Sivars U. Aivazian D. Pfeffer S. R. (2003). Yip3 catalyses the dissociation of endosomal Rab-GDI complexes. *Nature* 425 856–859. 10.1038/nature02057 14574414

[B64] Steger M. Tonelli F. Ito G. Davies P. Trost M. Vetter M. (2016). Phosphoproteomics reveals that Parkinson’s disease kinase LRRK2 regulates a subset of Rab GTPases. *Elife* 5:e12813 10.7554/eLife.12813 PMC4769169 26824392

[B65] Stroupe C. Brunger A. T. (2000). Crystal structures of a Rab protein in its inactive and active conformations. *J. Mol. Biol.* 304 585–598. 10.1006/jmbi.2000.4236 11099382

[B66] Tan E. K. Zhao Y. Skipper L. Tan M. G. Di Fonzo A. Sun L. (2007). The LRRK2 Gly2385Arg variant is associated with Parkinson’s disease: genetic and functional evidence. *Hum. Genet.* 120 857–863. 10.1007/s00439-006-0268-0 17019612

[B67] Valente E. M. Abou-Sleiman P. M. Caputo V. Muqit M. M. Harvey K. Gispert S. (2004). Hereditary early-onset Parkinson’s disease caused by mutations in PINK1. *Science* 304 1158–1160. 10.1126/science.1096284 15087508

[B68] Wang X. Yan M. H. Fujioka H. Liu J. Wilson-Delfosse A. Chen S. G. (2012). LRRK2 regulates mitochondrial dynamics and function through direct interaction with DLP1. *Hum. Mol. Genet.* 21 1931–1944. 10.1093/hmg/dds003 22228096 PMC3315202

[B69] Waschbusch D. Michels H. Strassheim S. Ossendorf E. Kessler D. Gloeckner C. J. (2014). LRRK2 transport is regulated by its novel interacting partner Rab32. *PLoS ONE* 9:e111632 10.1371/journal.pone.0111632 PMC4216093 25360523

[B70] Wilson G. R. Sim J. C. McLean C. Giannandrea M. Galea C. A. Riseley J. R. (2014). Mutations in RAB39B cause X-linked intellectual disability and early-onset Parkinson disease with alpha-synuclein pathology. *Am. J. Hum. Genet.* 95 729–735. 10.1016/j.ajhg.2014.10.015 25434005 PMC4259921

[B71] Wu X. S. Rao K. Zhang H. Wang F. Sellers J. R. Matesic L. E. (2002). Identification of an organelle receptor for myosin-Va. *Nat. Cell Biol.* 4 271–278. 10.1038/ncb760 11887186

[B72] Yamano K. Fogel A. I. Wang C. van der Bliek A. M. Youle R. J. (2014). Mitochondrial Rab GAPs govern autophagosome biogenesis during mitophagy. *Elife* 3:e01612 10.7554/eLife.01612 PMC3930140 24569479

[B73] Yin G. Lopes da Fonseca T. Eisbach S. E. Anduaga A. M. Breda C. Orcellet M. L. (2014). alpha-Synuclein interacts with the switch region of Rab8a in a Ser129 phosphorylation-dependent manner. *Neurobiol. Dis.* 70 149–161. 10.1016/j.nbd.2014.06.018 24983211

[B74] Zimprich A. Benet-Pages A. Struhal W. Graf E. Eck S. H. Offman M. N. (2011). A mutation in VPS35, encoding a subunit of the retromer complex, causes late-onset Parkinson disease. *Am. J. Hum. Genet.* 89 168–175. 10.1016/j.ajhg.2011.06.008 21763483 PMC3135812

[B75] Ziviani E. Tao R. N. Whitworth A. J. (2010). *Drosophila* parkin requires PINK1 for mitochondrial translocation and ubiquitinates mitofusin. *Proc. Natl. Acad. Sci. U.S.A.* 107 5018–5023. 10.1073/pnas.0913485107 20194754 PMC2841909

